# Decoration of PdAg Dual-Metallic Alloy Nanoparticles on Z-Scheme α-Fe_2_O_3_/CdS for Manipulable Products *via* Photocatalytic Reduction of Carbon Dioxide

**DOI:** 10.3389/fchem.2022.937543

**Published:** 2022-07-22

**Authors:** Shuhui Yang, Xi Ke, Menglong Zhang, Dongxiang Luo

**Affiliations:** ^1^ Institute of Semiconductors, South China Normal University, Guangzhou, China; ^2^ School of Chemistry and Chemical Engineering/Institute of Clean Energy and Materials/Guangzhou, Key Laboratory for Clean Energy and Materials/Huangpu Hydrogen Innovation Center, Guangzhou University, Guangzhou, China; ^3^ School of Materials and Energy, Guangdong University of Technology, Guangzhou, China

**Keywords:** composite materials, metal and alloys, photocatalysis, photoelectrochemistry, manipulable products

## Abstract

Metal nanoparticles have been extensively used as co-catalysts in photocatalytic systems in order to pursue improvements in both reaction kinetics and selectivity. In this work, PdAg dual-metallic nanoparticles synthesized by the co-reduction method were decorated on a well-established α-Fe_2_O_3_/CdS Z-scheme photoactive material as a co-catalyst to study their performance for promoting the photoreduction of CO_2_. Herein, α-Fe_2_O_3_ and CdS were *in situ* synthesized on fluorine-doped tin oxide (FTO) glass by hydrothermal and SILAR (successive ionic layer adsorption and reaction) methods, respectively. The direct Z-scheme charge transfer path between Fe_2_O_3_ and CdS and the effective electron migration toward the PdAg mainly contributed to the excellent photocatalytic CO_2_ reduction performance. The controllable work function based on Pd (5.12) and Ag (4.26) constructed an appropriate band alignment with α-Fe_2_O_3_/CdS and displayed favorable production for CH_4_ rather than CO. The optimum ratio of PdAg 1:2 performed a 48% enhancement than pure Pd for photoreduction of CO_2_. Meanwhile, the enhanced charge separation improved the photoelectrochemical performance and photocurrent generation, and reduced the electrical resistance between components. This work provided insights into the dual-metallic co-catalyst for boosting the activity and selectivity of photocatalytic CO_2_ reduction.

## Introduction

Solar-driven chemical reactions based on photoactive materials have attracted massive attention due to the concerns with regard to the current energy and environmental issues (Baharuddin et al., 2021). The fundamental research summarized some key features that were capable of improving the photoactive materials for practical solar-driven chemical reactions. These were broad light absorption range, rapid charge separation, good stability, abundant surface area, and reaction sites. Hematite (α-Fe_2_O_3_) was considered as an outstanding photocatalyst due to its good ability of light response and light harvesting (absorbs ca. 30% light) (Kuang et al., 2017; Yi et al., 2021). However, its short lifetime of photogenerated charge was attributed to the limited hole diffusion distance (Shen et al., 2016). In order to improve the charge migration rate of α-Fe_2_O_3_, a previous study focused on constructing heterostructures with different bandgap materials for further improving photogenerated charge separation (Kanwal et al., 2021). For instance, Zhang et al. reported that CdS/α-Fe_2_O_3_ heterojunction showed great photocatalytic activity and had a prominent charge separation rate due to the fast diffusion of photogenerated charge between Fe_2_O_3_ and CdS (Zhang et al., 2013). Based on previous studies, we considered an optimization where the role of CdS was to help Fe_2_O_3_ with a quick charge transfer except for light absorption. Previous research demonstrated that noble metal nanoparticles possessed good chemical stability, large surface area, and fruitful reaction sites. Among the heterogeneous metallic co-catalysts, Pd-based nanoparticles have presented an outstanding performance for promoting photocatalytic reactions under mild conditions (Naina et al., 2021; Peng et al., 2021; Cui et al., 2022). This is due to the intensive electronic interactions between metal nanoparticles and semiconductive photocatalysts (Mott–Schottky effect) (Li and Antonietti, 2013). In comparison to pure Pd, the formation of the PdAg alloy was found to enhance the catalytic performance by applying electronic and geometric effects (Antolini, 2009). Furthermore, the PdAg alloy with different lattice constants of the dual metal atoms (Ag, 408.53pm and Pd, 389.07pm) could subsequently improve the absorption of visible light and reaction rate due to the d-band shifting up (Ghiabi et al., 2018).

The photocatalytic reduction of CO_2_ into valuable solar fuels was regarded as an efficient means to address the energy supply and CO_2_ emission (Habisreutinger et al., 2013). In addition, using semiconductors such as hematite (α-Fe_2_O_3_) to achieve this conversion goal had attracted extensive attention, since they were an environmentally friendly low-cost technology (Mu et al., 2021). It is well-known that the main products *via* photoreduction of CO_2_ are CO and CH_4_. In addition, controlling the chemical reaction conditions (Qiao et al., 2014) (pH, temperature, sacrificial reagent, et al.) is the common means to manipulate the selectivity, although it might be complex.

**SCHEME 1:**
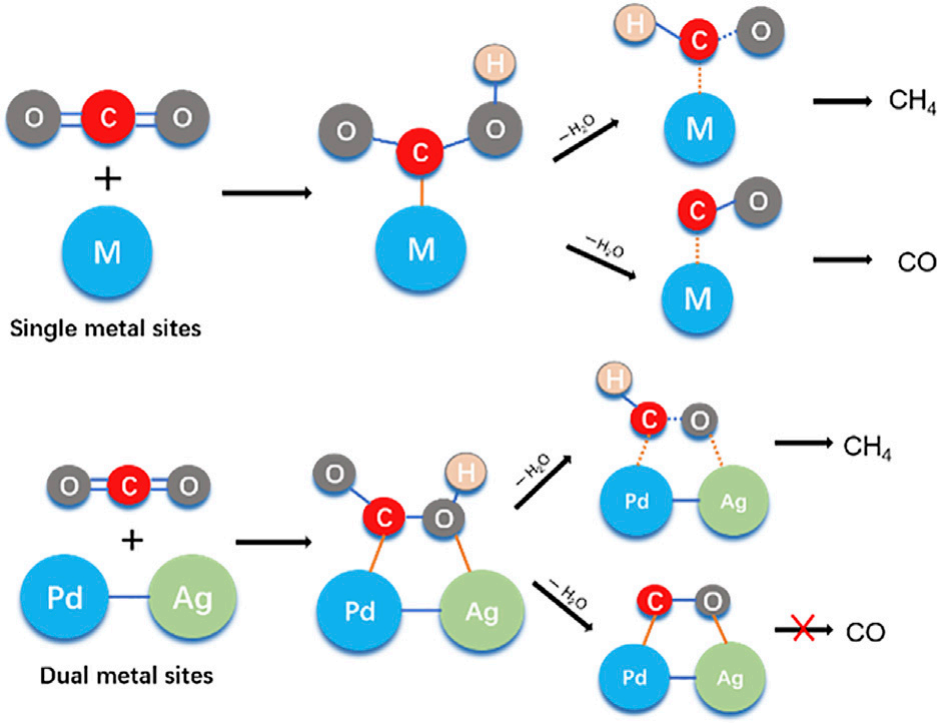
The modulated CO_2_ reduction process on single and dual metal sites.

However, a dual-metallic co-catalyst could be an alternative strategy to manipulate the selectivity of CO_2_ photoreduction, as recent modulating research (Li et al., 2019) demonstrated that the dual-metal sites can dissociate CO production. This is because the C and O atoms from the adsorbed CO_2_ species would coordinate with two individual metal atoms, leading to a configuration of M_1_-C-O-M_2_ (M stands for metal, as shown in Scheme 1). The robust stability of M_1_-C-O-M_2_ would inhibit the breaking of M-C or M-O bonds, and thus dissociate the yield of CO. More importantly, it would conduct rapid protonation of C atoms in M_1_-C-O-M_2_ intermediates and provide a reaction path to yield CH_4_ (Wang et al., 2020).

Of direct relevance to our work, PdAg NPs have been successfully applied to g-C_3_N_4_ (Liu et al., 2019), carbon tube (Benipal et al., 2017), and reduced graphene oxide (Martins et al., 2017) for photocatalytic reactions. These studies demonstrated that PdAg was capable of promoting photocatalytic reactions and focused on the role of PdAg under different reaction conditions. However, the Pd:Ag atomic ratio and its influence on the photocatalytic selectivity and activity was still unexplored. Thus, in this work, an α-Fe_2_O_3_/CdS Z-scheme photocatalyst was employed as a photoactive platform to support a layer of PdAg NPs. The impact of the elemental ratio of PdAg NPs on photoelectrochemical features and photoreduction selectivity over CO_2_ was experimentally investigated.

## Experiment

### Materials and Reagents

Iron chloride hexahydrate (FeCl_3_·6H_2_O, 99%, Aladdin), cadmium acetate dihydrate (Cd(Ac)_2_·2H_2_O, Macklin, AR), ethanol (CH_3_CH_2_OH, Macklin, 95%), sodium sulfide nonahydrate (Na_2_S·9H_2_O, Macklin, >98%), dimethyldioctadecylammonium chloride (DODAC, C_38_H_80_ClN, Aladdin, 97%), silver nitrate (AgNO_3_, Aladdin, 99.8%), palladium chloride (PdCl_2_, Aladdin, 99.999%), hydrochloric acid (HCl, Guangzhou Chemical Reagent Factory, 36–38%), polyvinylpyrrolidone (PVP, (C_6_H_9_NO)_n_, Aladdin), ascorbic acid (C_6_H_8_O_6_, Aladdin, 99%), and sodium sulfite (Na_2_SO_3_, Aladdin, 98.0%) were used. All the raw materials were used without further separation and purification.

### Synthesis of Fe_2_O_3_

The Fe_2_O_3_ nanorod arrays were prepared following a modified literature report (Ma et al., 2010). The cleaned FTO was entirely immersed in a 50-ml hydrothermal reactor with FeCl_3_ (0.15 M) aqueous solution and heated to 100°C for 6 h. After the hydrothermal reaction, the samples were collected and rinsed with deionized water and N_2_ stream drying. The sample was then annealed in a muffle furnace at 550°C for 2 h (10°C/min) and collected after cooling down to room temperature.

### Fabrication of Fe_2_O_3_/CdS

The electrode of Fe_2_O_3_ nanorods deposited by CdS was fabricated *via* the SILAR (successive ionic layer adsorption and reaction) method (Liu et al., 2013). The as-prepared Fe_2_O_3_ film was first soaked in Cd(Ac)_2_ (25 mM) ethanol solution for 1 min, dried by N_2_ stream. Next, it was immersed in Na_2_S (25 mM) aqueous solution for 1 min and then washed with deionized water, dried by N_2_ stream. The deposition of CdS was accomplished by following chemical reactions for 10 SILAR cycles. Then, the electrode was finally annealed under Ar stream at 400°C for 30 min (1°C/min).

### Fabrication of Fe_2_O_3_/CdS/PdAg

The preparation of PdAg was more complicated through the co-reduction of Pd and Ag using DODAC as a structure-directing agent (Zhou et al., 2020). AgNO_3_ (10 mM) and H_2_PdCl_4_ (20 mM, prepared from PdCl_2_ and HCl aqueous solution) were added to DODAC (2 mM) which was dissolved in deionized water. In the next step, PVP (10 mg/ml) was added for better dispersion. During the co-reduction process of Pd and Ag, ascorbic acid (0.3 M) aqueous solution was added with the color change (slight yellow to black) under 60°C for 30 min.

The dispersant absorption method was employed for the deposition of PdAg on Fe_2_O_3_/CdS heterojunction. To be specific, the as-prepared Fe_2_O_3_/CdS film was dipped into the PdAg solution for 1 min and then dried by N_2_ steam; this process was repeated 5 times. Then, the electrode decorated with Fe_2_O_3_/CdS/PdAg was annealed under an Ar stream at 500°C for 30 min (10°C/min). It was noted that the Pd:Ag ratio was controlled by the molar amount of each precursor.

### Characterization

The phase information was characterized by X-ray diffraction with Cu Kα radiation. The morphologies and structures of the ternary composites were observed on a scanning electron microscope (Hitachi S-4800 field emission SEM, Tokyo, Japan) and a transmission electron microscope (TEM, JEOL, JEM 2100), respectively. X-ray photoelectron spectroscopy (VG ESCALAB XPS System with a monochromatized Al Kα X-ray source) was carried out to investigate the chemical state. UV-vis diffuse reflectance spectroscopy and absorption spectroscopy equipped with a DH-2000-BAL lamp (deuterium/helium) of wavelength ranging from 200 to 1,100 nm were used to characterize the optical properties and bandgap. The photoreduction of CO_2_ was conducted in a home-made 100-ml Pyrex reactor with an optical window. The reactor was sealed using a rubber septum. A Xe lamp with 300 W and an AM 1.5-G filter were used as the simulated solar light. The samples were scraped from the FTO slides and sonicated for 1 h before the reaction. During the reaction, 1 ml of gas was acquired from the reactor at a specified interval (1 h), and subsequent gas concentration analysis was performed in a GC-2014C Shimazu equipped with a flame-ionization detector. The photoelectrochemical (PEC) performances were measured in an alkaline solution (0.25 M Na_2_S/0.35 M Na_2_SO_3_, pH = 13.1) using a standard three-electrode PEC cell, under the simulated sunlight (with AM 1.5-G filter). The sample, Ag/AgCl, and Pt wire were employed as the working electrode, reference electrode, and counter electrode, respectively. The potential vs. RHE was determined using the equation, from which the E(RHE) = E(Ag/AgCl) + 0.964 V (Mohamed et al., 2022)

E(RHE)=0.197+0.0591pH+E(Ag/AgCl).

## Result and Discussion

**FIGURE 1:**
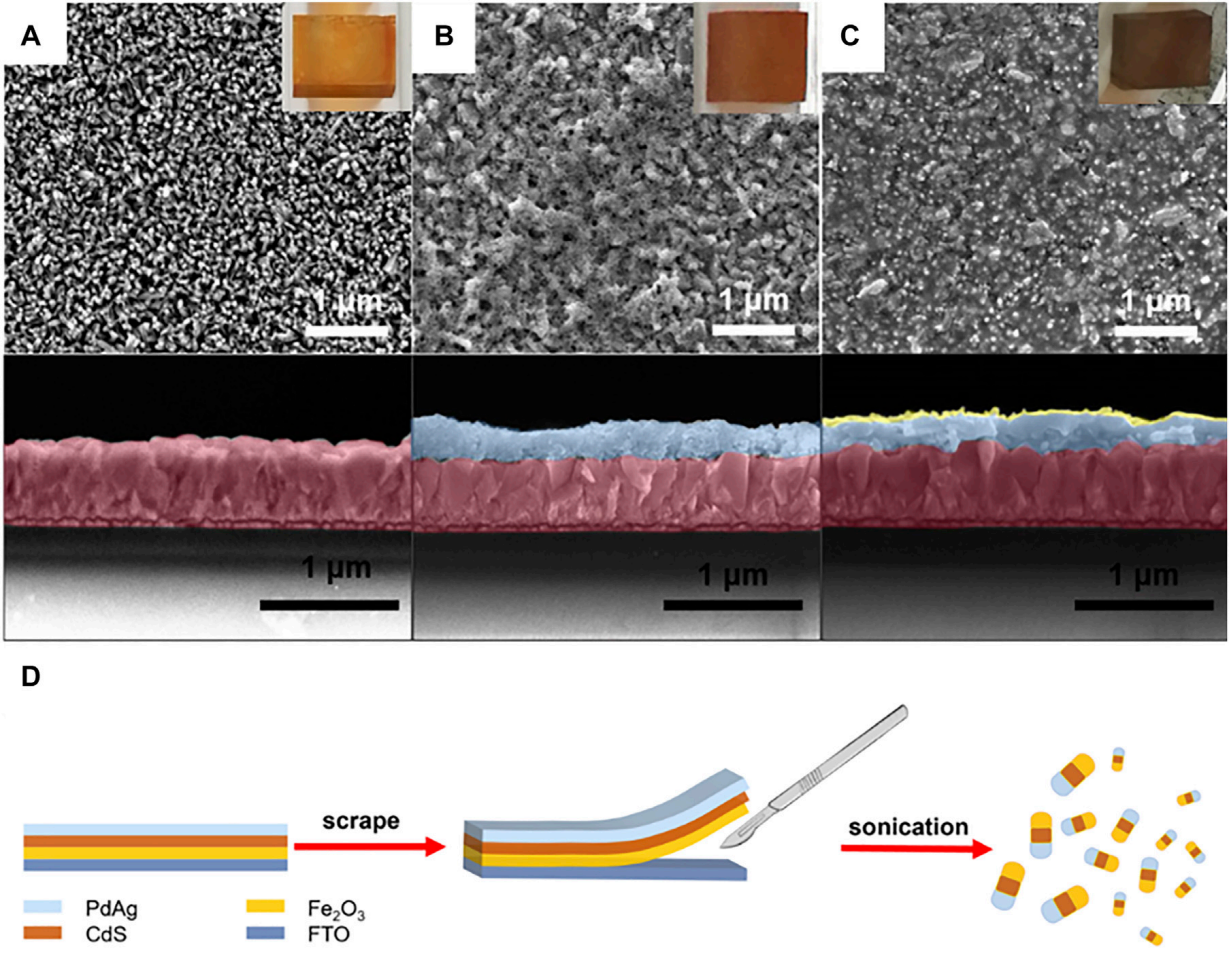
SEM images of the top view, cross-section, and digital photographs of **(A)** α-Fe_2_O_3_, **(B)** α-Fe_2_O_3_/CdS, **(C)** α-Fe_2_O_3_/CdS/PdAg on FTO substrates, and **(D)** schematic diagram of a process for preparing samples for CO_2_ reduction tests.

**FIGURE 2:**
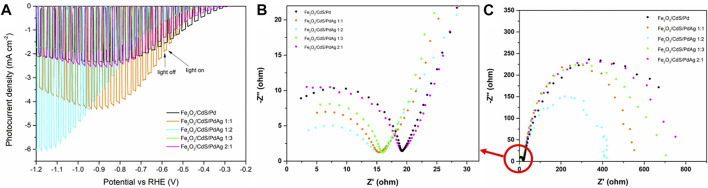
**(A)** Cathodic linear sweep voltammetry under chopped illumination; **(B)** and **(C)** electrochemical impedance spectroscopy (EIS) of samples with different Pd:Ag ratios.

The α-Fe_2_O_3_ film was first grown on the FTO glass substrate, which exhibited the nanorod morphology in SEM images (see supporting information in Supplementary Figure S1). In detail, the as-prepared film presented a light orange color due to the formation of FeOOH after hydrothermal reaction and then turned red after annealing under air at 500°C in a muffle furnace; the thickness of α-Fe_2_O_3_ was *ca*. 500 nm (Figure 2A) (Ahn et al., 2016; Yi et al., 2021). Subsequently, CdS was *in situ* synthesized on α-Fe_2_O_3_ using the SILAR method (Sankapal et al., 2000). The CdS with a thickness of *ca.* 270 nm (Figure 2B) was homogeneously coated on the α-Fe_2_O_3_ nanorod, and the α-Fe_2_O_3_/CdS heterojunction film presented a crimson color. Furthermore, PdAg alloy NPs were obtained through co-reduction of Pd and Ag precursors in an aqueous solution (Zhou et al., 2020). Then, a thin layer of PdAg 2:1 (the molar ratio between Pd and Ag is 2:1 in precursor) alloy NPs was coated above the CdS layer. The α-Fe_2_O_3_/CdS/PdAg on FTO was used as an electrode for photoelectrochemistry tests, and then Fe_2_O_3_/CdS/PdAg were scrapped from the substrate (Figure 1D) for gas chromatography tests. XRD patterns (Supplementary Figure S2A) of α-Fe_2_O_3_ and CdS were confirmed to be indexed to JCPDS No. 33-0664 and 41-1049, respectively. Moreover, the two components, Fe_2_O_3_ and CdS, were both in a hexagonal structure with slightly different lattice parameters (Zhang et al., 2013). PdAg NPs with an average particle size of 6.14 nm (calculated from 100 random particles) were found to be uniformly distributed on the α-Fe_2_O_3_/CdS (Supplementary Figure S2B). Furthermore, the spacing of the lattice fringe related to PdAg NPs was measured to be 2.48 nm between (111) the plane of Pd (2.245 nm) and (004) the plane of Ag (2.425 nm) (Gao et al., 2021) (Supplementary Figure S2C). The lattice fringes of α-Fe_2_O_3_ and CdS were recorded to be 0.251 and 0.273 nm, corresponding to the (110) and (200) planes, respectively. Meanwhile, it certified the weak diffraction peak of PdAg NPs in XRD patterns. From UV-vis diffusion reflectance spectroscopy (DRS), the light absorption of α-Fe_2_O_3_/CdS basically showed no differences after coupling with PdAg (Supplementary Figure S3). X-ray photoelectron spectroscopy (XPS) tested the Pd 3 d and Ag 3 d when PdAg was coupled with α-Fe_2_O_3_/CdS, which presented a slight shift toward higher binding energy (Supplementary Figure S4). Moreover, the pristine PdAg NPs were further qualitatively confirmed by energy-dispersive spectrum (EDS), where the atomic ratio between Pd and Ag in PdAg 2:1 was measured to be 62.1 and 37.9%, respectively (Supplementary Figure S2D). In addition, it is known that the successful connection between metal co-catalysts and photocatalysts would result in improved charge separation (Lan et al., 2021). Therefore, transient resolved photoluminescence (TRPL) was exploited to analyze the lifetime of charge after the decoration of PdAg, from which an enhanced lifetime (from 0.465 to 1.396 ns, Supplementary Figure S5) was observed, suggesting that the charge transfer was built between PdAg NPs and α-Fe_2_O_3_/CdS.

**FIGURE 3:**
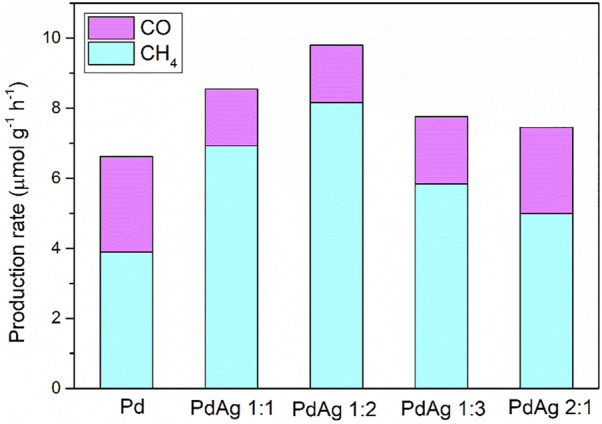
The production rate of photoreduction of CO_2_ over α-Fe_2_O_3_/CdS/PdAg of different Pd:Ag ratios.

The photoelectrochemical properties of α-Fe_2_O_3_, CdS, and α-Fe_2_O_3_/CdS were tested as an anodic electrode in a standard PEC cell. In accordance with a previous report (Shen et al., 2020), an improvement in the maximum photocurrent density and on-set potential was observed when coupling α-Fe_2_O_3_ with CdS (Supplementary Figure S6). Next, to evaluate the influence of PdAg on the photoelectrochemical features, α-Fe_2_O_3_/CdS/PdAg was set as the cathodic electrode in the PEC cell. Among samples, the higher Pd content led to a lower on-set potential to trigger the cathodic photocurrent generation (Figure 2A), due to the higher work function of Pd (5.12) than Ag (4.26). The largest photocurrent density was observed from the sample with PdAg 1:2. This sample also presented smaller radii as revealed by EIS tests (Figures 2B,C) compared to others, suggesting lower electronic resistance in favor of charge transfer and thus enhancing the photocurrent generation. In addition, the stability test was executed by electrochemical characterization. The relative photocurrent displayed a slight decrease (ca. 0.1) after 10 min of reaction in Supplementary Figure S7, which can be attributed to the consumption of sacrificial agents and impeded the photoactivity. Combining with the XRD data shown in Supplementary Figure S8, the position of each peak presented no obvious change before and after the reaction, which certified that the catalyst possessed good stability during the photocatalytic reactions. As for the CO_2_ reduction, gas chromatography (GC) confirmed the main products (CH_4_ and CO) from the samples decorated with different Pd:Ag ratios. As shown in Figure 3, the gas production rates of the samples were proportional to their photocurrent density in PEC tests. The highest production rate was observed from the sample with PdAg 1:2. Meanwhile, compared to the control with pure Pd (6.62 μmol g^−1^ h^−1^), PdAg 1:2 (9.80 μmol g^−1^ h^−1^) presented a 48% enhancement in the total conversion rate. More importantly, as the modulating research predicted, by controlling the Pd:Ag ratio, the selectivity toward CH_4_ was optimized from 58.7 to 83.2%.

**SCHEME 2:**
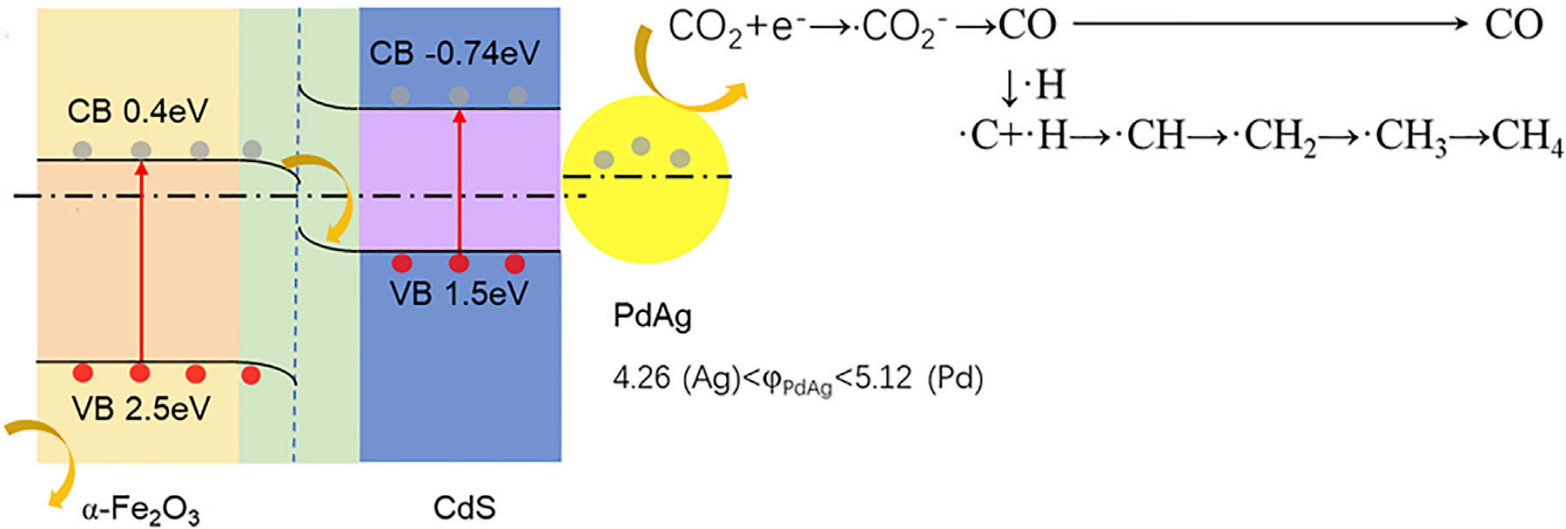
The band alignment of the α-Fe_2_O_3_/CdS/PdAg heterojunction and mechanism of photocatalytic CO_2_ reduction.

As shown in Scheme 2, an interfacial charge region was constructed *via* an α-Fe_2_O_3_/CdS heterojunction, and a CO_2_ reduction reaction occurred on the PdAg sites. In detail, the adsorbed CO_2_ reacted with electrons accumulated on the PdAg sites to form CO and CH_4_, respectively (Ješić et al., 2021). The advantage of this ternary structure was a synergic effect between the Z-scheme α-Fe_2_O_3_/CdS and co-catalyst PdAg, which helped in prompting the selectivity of CO and CH_4_.

## Conclusion

Overall, α-Fe_2_O_3_/CdS decorated by PdAg NPs of different Pd:Ag ratios was successfully prepared. The decoration of PdAg alloy NPs led to an enhanced photoreduction rate of CO_2_, but also ended up with an improvement in the selectivity toward CH_4_ production. The improved photocatalytic activity was contributed by the accumulation of electrons on the PdAg sides, which resulted in a 48% enhancement in comparison to the pure Pd (6.62 μmol g^−1^ h^−1^). On the other hand, the selectivity was attributed to the dual-metallic active sites supplied by PdAg NPs, which conducted continuous protonation of C atoms in M_1_-C-O-M_2_ intermediates. As a result, the sample with a ratio of PdAg 1:2 was in favor of producing CH_4_ instead of CO, which was consistent with the best photocurrent and smallest radii.

## Data Availability

The original contributions presented in the study are included in the article/Supplementary Material; further inquiries can be directed to the corresponding authors.

## References

[B1] Ahn H.-J. Yoon K.-Y. Kwak M.-J. Jang J.-H. (2016). A Titanium-Doped SiOxPassivation Layer for Greatly Enhanced Performance of a Hematite-Based Photoelectrochemical System. Angew. Chem. Int. Ed. 55, 9922–9926. 10.1002/anie.201603666 27358249

[B2] Antolini E. (2009). Palladium in Fuel Cell Catalysis. Energy Environ. Sci. 2, 915–931. 10.1039/b820837a

[B3] Baharuddin N. A. Wan Yusoff W. N. A. Abd Aziz A. J. Mohd Tahir N. N. (2021). Hydrogen Fuel Cells for Sustainable Energy: Development and Progress in Selected Developed Countries. IOP Conf. Ser. Mater. Sci. Eng. 1078 (1), 012011. 10.1088/1757-899x/1078/1/012011

[B4] Benipal N. Qi J. Liu Q. Li W. (2017). Carbon Nanotube Supported PdAg Nanoparticles for Electrocatalytic Oxidation of Glycerol in Anion Exchange Membrane Fuel Cells. Appl. Catal. B Environ. 210, 121–130. 10.1016/j.apcatb.2017.02.082

[B5] Cui C. Zhang Y. Shan W. Yu Y. He H. (2022). Influence of NO on the Activity of Pd/θ-Al2O3 Catalyst for Methane Oxidation: Alleviation of Transient Deactivation. J. Environ. Sci. 112, 38–47. 10.1016/j.jes.2021.04.020 34955221

[B6] Gao J. Zhang F. Xue H. Zhang L. Peng Y. Li X. (2021). In-situ Synthesis of Novel Ternary CdS/PdAg/g-C3N4 Hybrid Photocatalyst with Significantly Enhanced Hydrogen Production Activity and Catalytic Mechanism Exploration. Appl. Catal. B Environ. 281, 119509. 10.1016/j.apcatb.2020.119509

[B7] Ghiabi C. Ghaffarinejad A. Kazemi H. Salahandish R. (2018). *In Situ*, One-Step and Co-Electrodeposition of Graphene Supported Dendritic and Spherical Nano-Palladium-Silver Bimetallic Catalyst on Carbon Cloth for Electrooxidation of Methanol in Alkaline Media. Renew. Energy 126, 1085–1092. 10.1016/j.renene.2018.04.040

[B8] Habisreutinger S. N. Schmidt-Mende L. Stolarczyk J. K. (2013). Photocatalytic Reduction of CO2on TiO2and Other Semiconductors. Angew. Chem. Int. Ed. 52, 7372–7408. 10.1002/anie.201207199 23765842

[B9] Ješić D. Lašič Jurković D. Pohar A. Suhadolnik L. Likozar B. (2021). Engineering Photocatalytic and Photoelectrocatalytic CO2 Reduction Reactions: Mechanisms, Intrinsic Kinetics, Mass Transfer Resistances, Reactors and Multi-Scale Modelling Simulations. Chem. Eng. J. 407, 126799. 10.1016/j.cej.2020.126799

[B10] Kanwal A. Sajjad S. Leghari S. A. K. Yousaf Z. (2021). Cascade Electron Transfer in Ternary CuO/α-Fe2O3/γ-Al2O3 Nanocomposite as an Effective Visible Photocatalyst. J. Phys. Chem. Solids 151, 109899. 10.1016/j.jpcs.2020.109899

[B11] Kuang Y. Yamada T. Domen K. (2017). Surface and Interface Engineering for Photoelectrochemical Water Oxidation. Joule 1, 290–305. 10.1016/j.joule.2017.08.004

[B12] Lan D. Pang F. Ge J. (2021). Enhanced Charge Separation in NiO and Pd Co-modified TiO2 Photocatalysts for Efficient and Selective Photoreduction of CO2. ACS Appl. Energy Mat. 4, 6324–6332. 10.1021/acsaem.1c01144

[B13] Li X.-H. Antonietti M. (2013). Metal Nanoparticles at Mesoporous N-Doped Carbons and Carbon Nitrides: Functional Mott-Schottky Heterojunctions for Catalysis. Chem. Soc. Rev. 42, 6593–6604. 10.1039/c3cs60067j 23765224

[B14] Li X. Sun Y. Xu J. Shao Y. Wu J. Xu X. (2019). Selective Visible-Light-Driven Photocatalytic CO2 Reduction to CH4 Mediated by Atomically Thin CuIn5S8 Layers. Nat. Energy 4, 690–699. 10.1038/s41560-019-0431-1

[B15] Liu H. Liu X. Yang W. Shen M. Geng S. Yu C. (2019). Photocatalytic Dehydrogenation of Formic Acid Promoted by a Superior PdAg@g-C3N4 Mott-Schottky Heterojunction. J. Mat. Chem. A 7, 2022–2026. 10.1039/c8ta11172c

[B16] Liu Z. Wang Y. Wang B. Li Y. Liu Z. Han J. (2013). PEC Electrode of ZnO Nanorods Sensitized by CdS with Different Size and its Photoelectric Properties. Int. J. Hydrogen Energy 38, 10226–10234. 10.1016/j.ijhydene.2013.06.028

[B17] Ma J. Lian J. Duan X. Liu X. Zheng W. (2010). α-Fe2O3: Hydrothermal Synthesis, Magnetic and Electrochemical Properties. J. Phys. Chem. C 114, 10671–10676. 10.1021/jp102243g

[B18] Martins M. Šljukić B. Metin Ö. Sevim M. Sequeira C. A. C. Şener T. (2017). Bimetallic PdM (M = Fe, Ag, Au) Alloy Nanoparticles Assembled on Reduced Graphene Oxide as Catalysts for Direct Borohydride Fuel Cells. J. Alloys Compd. 718, 204–214. 10.1016/j.jallcom.2017.05.058

[B19] Mohamed A. G. A. Zhou E. Zeng Z. Xie J. Gao D. Wang Y. (2022). Asymmetric Oxo-Bridged ZnPb Bimetallic Electrocatalysis Boosting CO2 -To-HCOOH Reduction. Adv. Sci. (Weinh) 9, e2104138. 10.1002/advs.202104138 34761550 PMC8811806

[B20] Mu Z. Chen S. Wang Y. Zhang Z. Li Z. Xin B. (2021). Controlled Construction of Copper Phthalocyanine/α‐Fe2O3 Ultrathin S‐Scheme Heterojunctions for Efficient Photocatalytic CO2 Reduction under Wide Visible‐Light Irradiation. Small Sci. 1, 2100050. 10.1002/smsc.202100050 PMC11935974 40213775

[B21] Naina V. R. Wang S. Sharapa D. I. Zimmermann M. Hähsler M. Niebl-Eibenstein L. (2021). Shape-Selective Synthesis of Intermetallic Pd3Pb Nanocrystals and Enhanced Catalytic Properties in the Direct Synthesis of Hydrogen Peroxide. ACS Catal. 11, 2288–2301. 10.1021/acscatal.0c03561

[B22] Peng L. Tian H. Cui X. Su L. Meng G. Ma Z. (2021). Dual Synergetic Catalytic Effects Boost Hydrogen Electric Oxidation Performance of Pd/W18O49. Nano Res. 14, 2441–2450. 10.1007/s12274-020-3248-0

[B23] Qiao J. Liu Y. Hong F. Zhang J. (2014). A Review of Catalysts for the Electroreduction of Carbon Dioxide to Produce Low-Carbon Fuels. Chem. Soc. Rev. 43, 631–675. 10.1039/c3cs60323g 24186433

[B24] Sankapal B. R. Mane R. S. Lokhande C. D. (2000). Successive Ionic Layer Adsorption and Reaction (SILAR) Method for the Deposition of Large Area (∼10 Cm2 Disulfide (SnS2) Thin Films. Mater. Res. Bull. 35 (12), 2027–2035. 10.1016/s0025-5408(00)00405-0

[B25] Shen R. Zhang L. Chen X. Jaroniec M. Li N. Li X. (2020). Integrating 2D/2D CdS/α-Fe2O3 Ultrathin Bilayer Z-Scheme Heterojunction with Metallic β-NiS Nanosheet-Based Ohmic-Junction for Efficient Photocatalytic H2 Evolution. Appl. Catal. B Environ. 266, 118619. 10.1016/j.apcatb.2020.118619

[B26] Shen S. Lindley S. A. Chen X. Zhang J. Z. (2016). Hematite Heterostructures for Photoelectrochemical Water Splitting: Rational Materials Design and Charge Carrier Dynamics. Energy Environ. Sci. 9, 2744–2775. 10.1039/c6ee01845a

[B27] Wang J. Lin S. Tian N. Ma T. Zhang Y. Huang H. (2020). Nanostructured Metal Sulfides: Classification, Modification Strategy, and Solar‐Driven CO2 Reduction Application. Adv. Funct. Mater. 31, 2008008. 10.1002/adfm.202008008

[B28] Yi S.-S. Wang Z.-Y. Li H.-M. Zafar Z. Zhang Z.-T. Zhang L.-Y. (2021). Coupling Effects of Indium Oxide Layer on Hematite Enabling Efficient Photoelectrochemical Water Splitting. Appl. Catal. B Environ. 283, 119649. 10.1016/j.apcatb.2020.119649

[B29] Zhang S. Xu W. Zeng M. Li J. Xu J. Wang X. (2013). Hierarchically Grown CdS/α-Fe2O3 Heterojunction Nanocomposites with Enhanced Visible-Light-Driven Photocatalytic Performance. Dalton Trans. 42, 13417–13424. 10.1039/c3dt51492g 23892325

[B30] Zhou Y. Zhou R. Zhu X. Han N. Song B. Liu T. (2020). Mesoporous PdAg Nanospheres for Stable Electrochemical CO2 Reduction to Formate. Adv. Mater 32, e2000992. 10.1002/adma.202000992 32538508

