# Supplementary material for: Decoration of PdAg Dual-Metallic Alloy Nanoparticles on Z-Scheme α-Fe2O3/CdS for Manipulable Products via Photocatalytic Reduction of Carbon Dioxide
Source: Front Chem. 2022 Jul 22;10:937543. doi: 10.3389/fchem.2022.937543 (PMC9353514; doi:10.3389/fchem.2022.937543)
Supplement: Supplementary file 1 [file DataSheet1.PDF]

Decoration of PdAg dual-metallic alloy nanoparticles on Z-scheme α-Fe_2O_3/CdS for manipulable products via photocatalytic reduction of carbon dioxide

Shuhui Yang[1], Xi Ke[1], Kang Wang[1], Menglong Zhang[1*] and Dongxiang Luo[23*]

[1]Institute of Semiconductors, South China Normal University, Guangzhou, 510006, P.R. China

[2]School of Chemistry and Chemical Engineering/Institute of Clean Energy and Materials/Guangzhou Key Laboratory for Clean Energy and Materials/Huangpu Hydrogen Innovation Center, Guangzhou University, Guangzhou 510006, PR China

[3]School of Materials and Energy, Guangdong University of Technology, 510006, P.R. China

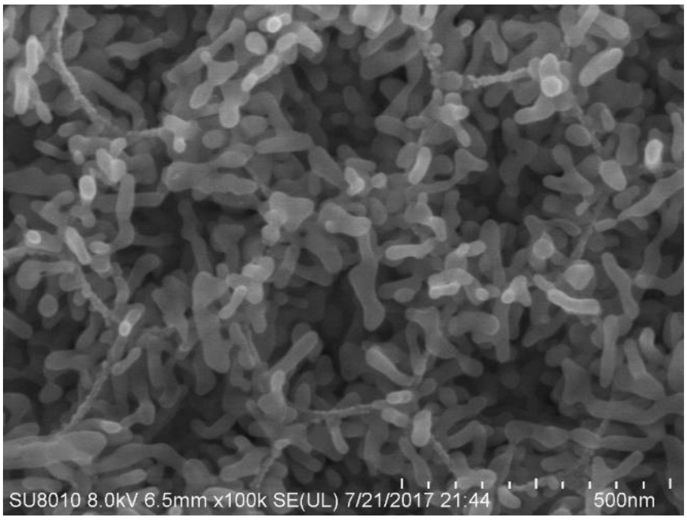

Figure S1. SEM image of α-Fe_2O_3 nanorod.

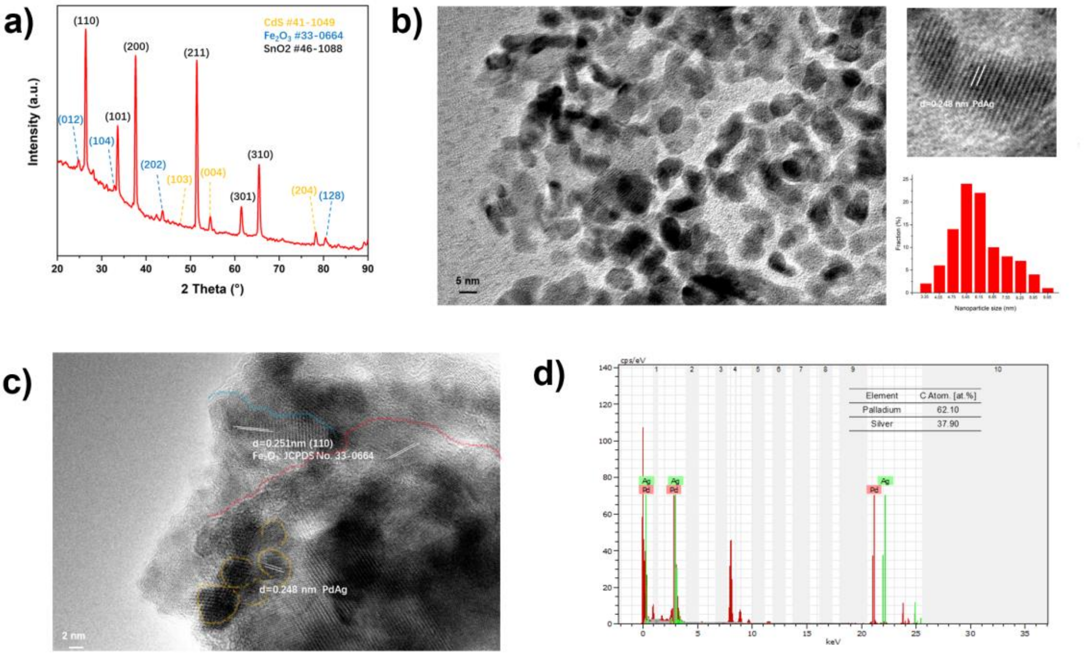

Figure S2. a) PXRD patterns of α-Fe_2O_3/CdS/PdAg 1:2; b) TEM image of pristine PdAg NPs and illustration of lattice fringe, statistical distribution of PdAg NPs size; c) TEM image of ternary composites α-Fe_2O_3/CdS/PdAg scraped from a slide; d) EDS spectrum of pristine PdAg NPs, in which red for Pd peak and green for Ag peak. The PXRD patterns and lattice fringes are corresponding to JCPDS 46-1088 (FTO), 33-0664 (α-Fe_2O_3), and 41-1049 (CdS).

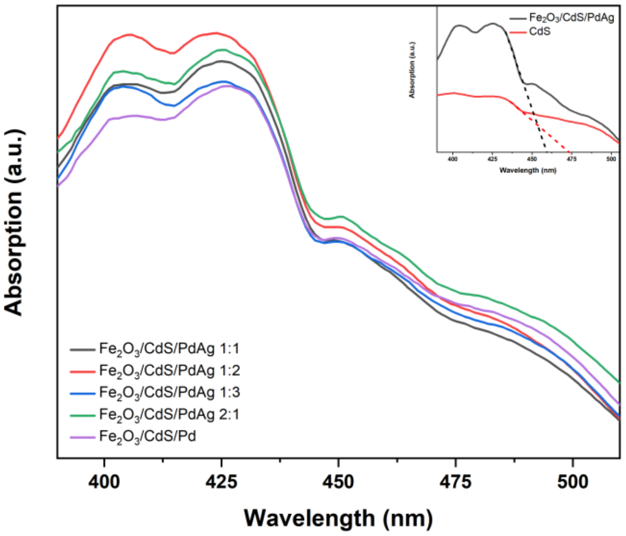

Figure S3. UV-vis spectrum of series photocatalysts.

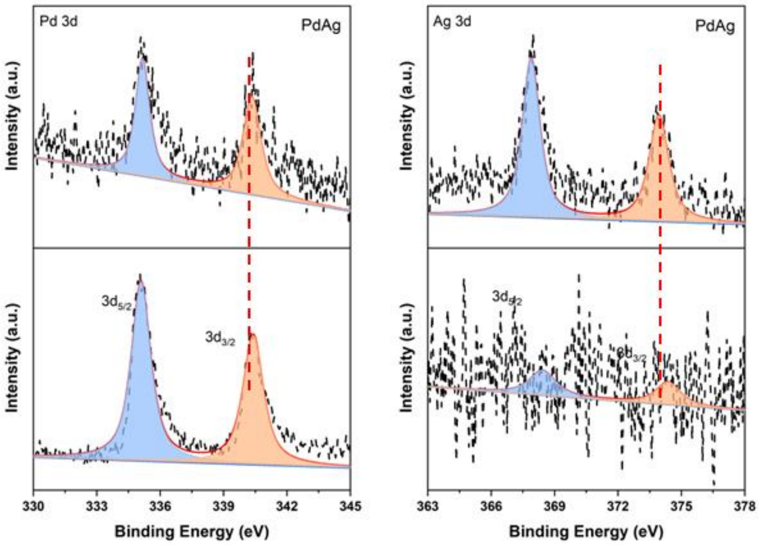

Figure S4. XPS spectrum of Pd 3d and Ag 3d in PdAg and α-Fe_2O_3/CdS/PdAg.

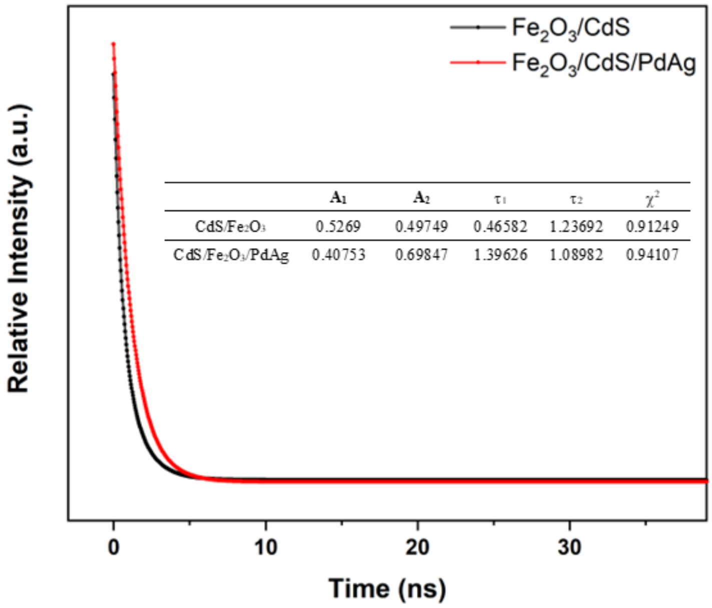

	A_1	A_2	τ_1	τ_2	χ^2
CdS/Fe_2O_3	0.5269	0.49749	0.46582	1.23692	0.91249
CdS/Fe_2O_3/PdAg	0.40753	0.69847	1.39626	1.08982	0.94107

Figure S5. TRPL spectra of α-Fe_2O_3/CdS and α-Fe_2O_3/CdS/PdAg (2:1).

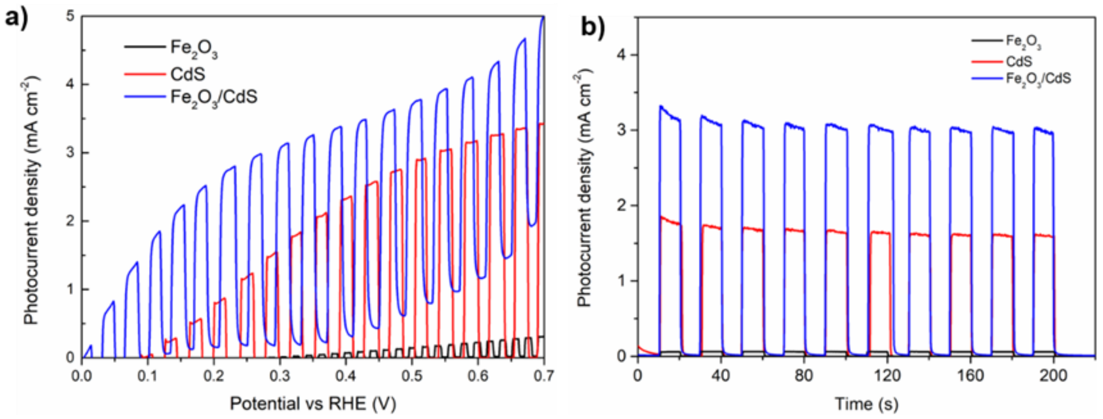

Figure S6. a) Linear sweep voltammetry and b) chronoamperometry at 0.3 V vs RHE of α-Fe_2O_3, CdS and α-Fe_2O_3/CdS.

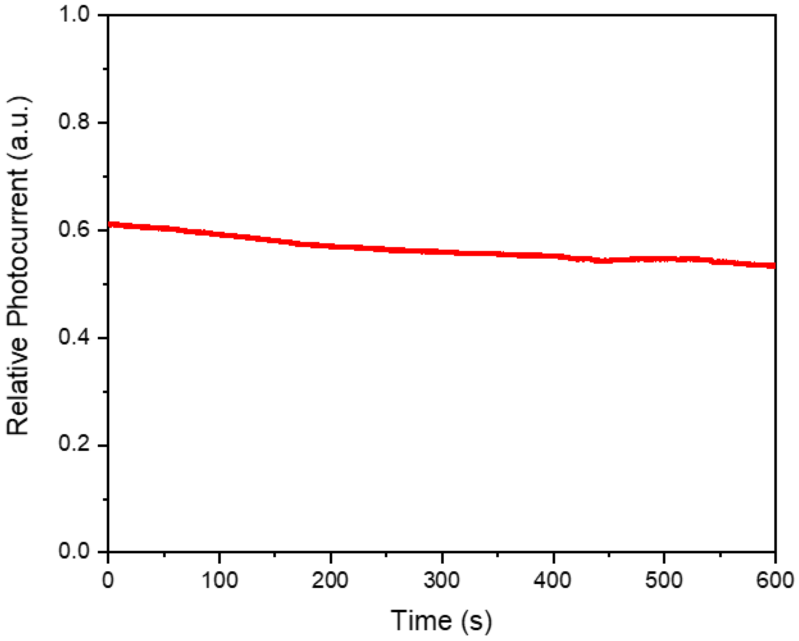

Figure S7. Relative photocurrent change at -0.3 V vs RHE of α-Fe_2O_3/CdS/PdAg 1:2 in 10 min.

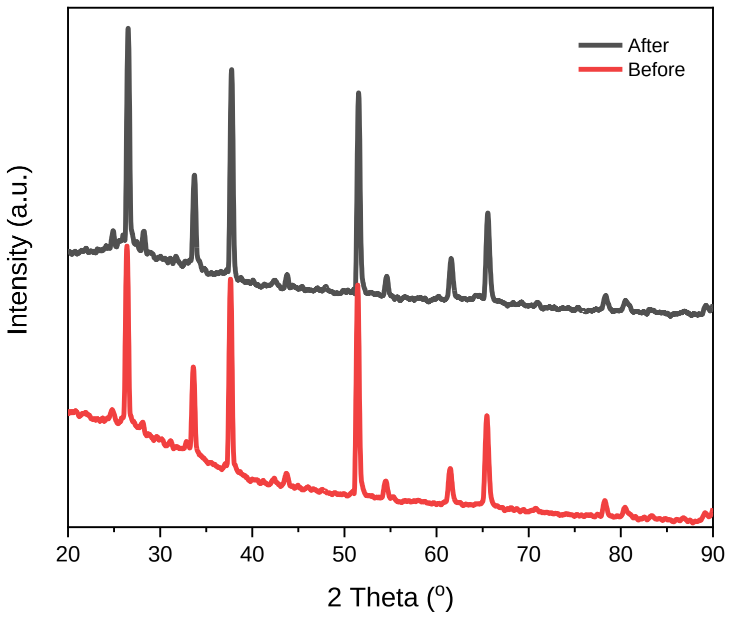

Figure S8. PXRD patterns of α-Fe_2O_3/CdS/PdAg 1:2 before and after electrochemical reaction.
